# Analysis of Trending Topics in Breast Cancer Articles From an Altmetric Perspective

**DOI:** 10.7759/cureus.26565

**Published:** 2022-07-05

**Authors:** Bahattin Bayar, Rıfat Peksöz

**Affiliations:** 1 General Surgery, Polatlı Duatepe State Hospital, Ankara, TUR; 2 General Surgery, Atatürk University Faculty of Medicine, Erzurum, TUR

**Keywords:** trend topics, citation, altmetric attention score, social media, breast cancer

## Abstract

Background

It is widely known that social media has an impact on politics and the economy. The Altmetric Attention Score (AAS) is a new Web-based metric that was recently developed for use in the scientific field. The objective of this study was to assess which recent studies on the topic of breast cancer received the most attention from the general public.

Methodology

An Altmetric Explorer search was performed on January 7, 2022, to extract the following information: journal name, impact factor (IF), year of publication, article topic, article type, and level of evidence.

Results

The journal that published articles that received the most attention on social media was the New England Journal of Medicine (n = 8). All of the articles were published in journals in the highest IF quartile. The most frequent top three subjects in the top 50 articles were “treatment and management,” “risk factors for breast cancer,” and “breast cancer screening.” The number of articles with a level of evidence of 1, 2, 3, and 4 was 12, 17, 17, and 4, respectively. The correlation between AAS and citation was not significant.

Conclusions

The AAS seems to be a more reliable assessment of public perception of breast cancer. We propose that combining the AAS and traditional metrics may provide a more detailed description of scientific research output.

## Introduction

The number of times published articles are cited is a common metric for determining a journal’s impact. Researchers use citation analysis (bibliometric analysis) to identify the most valuable publications in their fields, such as oncology [1], ophthalmology [2], and cardiology [3]. Eugene Garfield, the founder of the Institute of Scientific Information, was the first to apply this method to scientific publications in the 1970s [4]. Although citation analysis is a frequently used tool, its most important disadvantage is that the length of time that has passed since publication may have an impact on the total number of citations (length time bias). Additionally, citations only reflect the impact on the scientific community but do not show the impact on politicians, patients, or the general public.

Over the last few years, the importance of social media platforms in the promotion, dissemination, and presentation of medical literature has been markedly enhanced. Fast and dynamic assessments of the influence on social media platforms have been facilitated by a new Web-based metric (Altmetrics). The Altmetric Attention Score (AAS) is a quantitative and qualitative measurement that complements standard citation-based measures. It analyzes the interactions of academics, scholars, and scientists as captured by reference management tools and social media platforms such as Facebook, Twitter, LinkedIn, and blogs. The purpose of this research was to determine which recent breast cancer papers had the highest AAS.

## Materials and methods

Search engine

Altmetric Explorer (London, UK) is an internet-based tool that searches various sources of research output (listed in Table 1) to produce the most relevant and up-to-date picture of the different types of online activity and discussion [5].

**Table 1:** Altmetric Explorer (London, UK) sources.

Altmetric Explorer sources
1. Public policy documents
2. Blogs. There are >9,000 academic and non-academic blogs
3. Mainstream media. There are >4,000 outlets worldwide
4. Citations
5. Online reference managers
6. Research highlights
7. Post-publication peer-review platforms such as Pubpeer and Publons
8. Social media platforms, such as Facebook, Twitter, LinkedIn, Google+, Sina Weibo, and Pinterest
9. Wikipedia (tracks the 12 different language versions of Wikipedia)
10. Open Syllabus Project, which involves >4,000 institutions worldwide
11. Multimedia and other online platforms, such as YouTube, Reddit, and Q&A
12. Patents using data from IFI CLAIMS^®^

**Figure 1:**
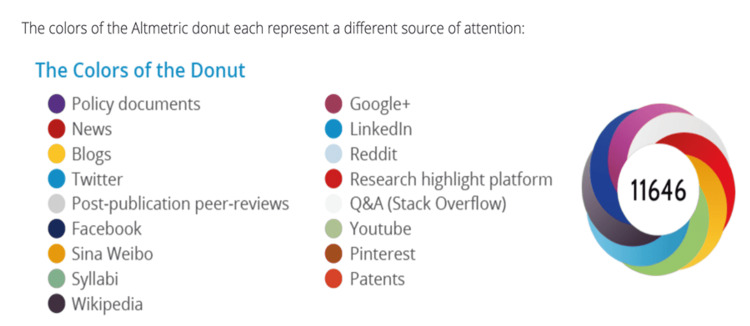
Altmetric donut.

The AAS is generated automatically using a weighted count of all the attention given to research output. The Altmetric donut and the AAS are designed to make determining how much and what type of attention a particular research product has received easier [6]. Volume, sources, and authors are the three main components. The Altmetric donut is colored to represent a different source of attention (Figure 1) [6].

Search strategy

**Figure 2:**
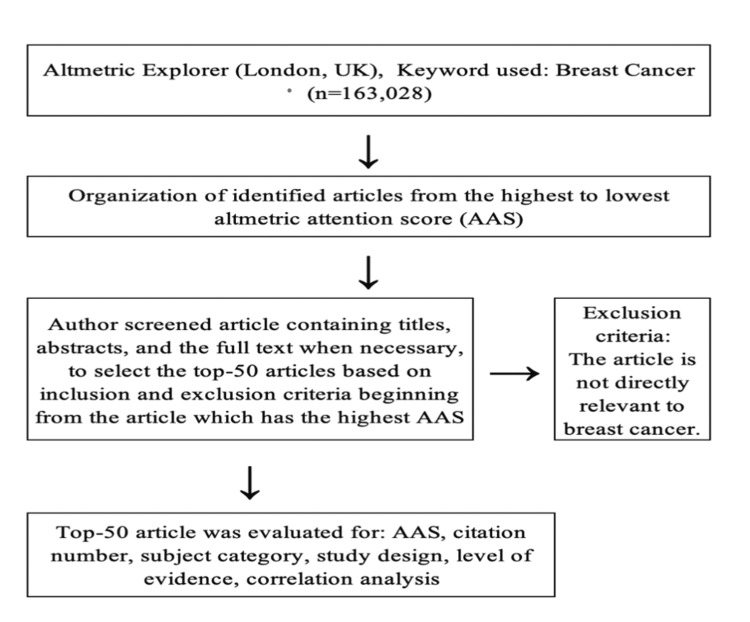
Flowchart illustrating the article allocation process.

An Altmetric Explorer search was performed on January 7, 2022. The term “breast cancer” was searched on the Altmetric Explorer (Figure 2). To clarify the AAS for the breast cancer field, the top 50 articles with the highest AAS were identified by excluding the articles that were not directly related to breast cancer. The data were then further evaluated by examining the title, journal name, date of publication, study type, and topic. On the same day, the number of citations for each article was acquired from the Thomson Reuters Web of Science citation-indexing database. Because altmetrics started to obtain data in 2011, in the search list, publications dated before 2011 were not included. The level of evidence of the top 100 cited articles was detected in accordance with the Scottish Intercollegiate Guidelines Network (SIGN) criteria [7]. The level of evidence was assigned to studies based on the methodological quality of their design, validity, and applicability to patient care.

Continuous variables were defined using median and interquartile range (IQR), whereas categorical variables were defined using percentages. The Kruskal-Wallis test was applied to compare three or more groups because the data were not normally distributed. The Spearman correlation coefficient was used for assessing the correlation between AAS, citations, number of years post-publication. P < 0.05 was considered statistically significant. All statistical analyses were performed using SPSS Statistics for Windows, version 21.0 (IBM Corp., Armonk, NY, USA).

## Results

We identified 163,028 articles regarding breast cancer using Altmetric Explorer. Table 2 summarizes the AAS, journal name, publication year, and citation count of the top 50 publications. Because altmetrics began collecting data in 2011, no articles prior to 2011 are listed in Table 2.

**Table 2:** Top 50 articles with the highest Altmetric Attention Score. JAMA: Journal of the American Medical Association; JNCI: Journal of the National Cancer Institute

Rank	Article title	Journal name	Date of publication	Altmetric Attention Score	Number of citations
1	International evaluation of an AI system for breast cancer screening	Nature	January 2020	3,619	443
2	Adjuvant chemotherapy guided by a 21-gene expression assay in breast cancer	New England Journal of Medicine	July 2018	2,912	788
3	Hair dye and chemical straightener use and breast cancer risk in a large US population of black and white women	International Journal of Cancer	December 2019	2,709	22
4	Immune recognition of somatic mutations leading to complete durable regression in metastatic breast cancer	Nature Medicine	June 2018	2,279	335
5	Pigeons (Columba livia) as trainable observers of pathology and radiology breast cancer images	Plos One	November 2015	1,890	41
6	Dietary supplement use during chemotherapy and survival outcomes of patients with breast cancer enrolled in a Cooperative Group Clinical Trial (SWOG S0221)	Journal of Clinical Oncology	March 2020	1,682	48
7	Breast-cancer tumor size, overdiagnosis, and mammography screening effectiveness	New England Journal of Medicine	October 2016	1,681	310
8	Contemporary hormonal contraception and the risk of breast cancer	New England Journal of Medicine	December 2017	1,655	180
9	Prospective validation of a 21-gene expression assay in breast cancer	New England Journal of Medicine	November 2015	1,544	769
10	High proliferation rate and a compromised spindle assembly checkpoint confers sensitivity to the MPS1 inhibitor BOS172722 in triple-negative breast cancers	Molecular Cancer Therapeutics	October 2019	1,531	6
11	Mediterranean diet and invasive breast cancer risk among women at high cardiovascular risk in the PREDIMED trial	JAMA Internal Medicine	December 2015	1,446	227
12	Type and timing of menopausal hormone therapy and breast cancer risk: individual participant meta-analysis of the worldwide epidemiological evidence	The Lancet	September 2019	1,438	176
13	Sustained weight loss and risk of breast cancer in women 50 years and older: a pooled analysis of prospective data	JNCI	December 2019	1,381	16
14	Breast cancer screening for women at average risk	JAMA	October 2015	1,351	817
15	Effect of mammographic screening from age 40 years on breast cancer mortality (UK Age trial): final results of a randomised, controlled trial	Lancet Oncology	September 2020	1,340	35
16	Glyphosate induces human breast cancer cells growth via estrogen receptors	Food & Chemical Toxicology	September 2013	1,331	191
17	Targeting the cancer mutanome of breast cancer	Nature Medicine	June 2018	1,285	5
18	National expenditure for false-positive mammograms and breast cancer overdiagnoses estimated at $4 billion a year	Health Affairs	April 2015	1,269	63
19	Effect of mistimed eating patterns on breast and prostate cancer risk (MCC-Spain Study )	International Journal of Cancer	July 2018	1,224	22
20	Breast cancer screening using tomosynthesis in combination with digital mammography	JAMA	June 2014	1,223	504
21	Mutant p53 drives the loss of heterozygosity by the upregulation of Nek2 in breast cancer cells	Breast Cancer Research	December 2020	1,158	0
22	70-gene signature as an aid to treatment decisions in early-stage breast cancer	New England Journal of Medicine	August 2016	1,148	842
23	Asparagine bioavailability governs metastasis in a model of breast cancer	Nature	February 2018	1,101	190
24	Long term survival and local control outcomes from single dose targeted intraoperative radiotherapy during lumpectomy (TARGIT-IORT) for early breast cancer: TARGIT-A randomised clinical trial	British Medical Journal	August 2020	1,077	40
25	Breast cancer risk from modifiable and nonmodifiable risk factors among white women in the United States	JAMA Oncology	October 2016	1,074	157
26	Honeybee venom and melittin suppress growth factor receptor activation in HER2-enriched and triple-negative breast cancer	npj Precision Oncology	September 2020	1,052	22
27	Menopausal hormone therapy and 20-year breast cancer mortality	The Lancet	September 2019	1,049	21
28	Atezolizumab and nab-paclitaxel in advanced triple-negative breast cancer	New England Journal of Medicine	November 2018	1,046	1562
29	Twenty five year follow-up for breast cancer incidence and mortality of the Canadian National Breast Screening Study: randomised screening trial	British Medical Journal	February 2014	1,012	252
30	Hair product use and breast cancer risk among African American and White women	Carcinogenesis	June 2017	1,012	25
31	Fine-mapping of 150 breast cancer risk regions identifies 191 likely target genes	Nature Genetics	January 2020	1,007	38
32	Dietary isoflavone intake and all-cause mortality in breast cancer survivors: The Breast Cancer Family Registry	Cancer	March 2017	998	35
33	Association of body mass index and age with subsequent breast cancer risk in premenopausal women	JAMA Oncology	November 2018	997	117
34	Breast cancer screening in Denmark	Annals of Internal Medicine	January 2017	996	80
35	Breast cancer statistics, 2017, racial disparity in mortality by state	CA: A Cancer Journal for Clinicians	October 2017	981	933
36	Gain fat—lose metastasis: converting invasive breast cancer cells into adipocytes inhibits cancer metastasis	Cancer Cell	January 2019	967	99
37	Usual consumption of specific dairy foods is associated with breast cancer in the Roswell Park Cancer Institute Data Bank and BioRepository	Current Developments in Nutrition	February 2017	942	6
38	Association between use of a scalp cooling device and alopecia after chemotherapy for breast cancer	JAMA	February 2017	936	71
39	Breast cancer screening, incidence, and mortality across US counties	JAMA Internal Medicine	September 2015	923	128
40	Landscape of somatic mutations in 560 breast cancer whole-genome sequences	Nature	May 2016	922	1012
41	COVID-19 impact on screening test volume through the National Breast and Cervical Cancer early detection program, January–June 2020, in the United States	Preventive Medicine	October 2021	919	4
42	Dairy, soy, and risk of breast cancer: those confounded milks	International Journal of Epidemiology	February 2020	917	20
43	Postdiagnosis social networks and breast cancer mortality in the After Breast Cancer Pooling Project	Cancer	December 2016	913	35
44	Assessment of machine learning of breast pathology structures for automated differentiation of breast cancer and high-risk proliferative lesions	JAMA Network Open	August 2019	907	17
45	30-day mortality after systemic anticancer treatment for breast and lung cancer in England: a population-based, observational study	Lancet Oncology	September 2016	906	83
46	Fasting mimicking diet as an adjunct to neoadjuvant chemotherapy for breast cancer in the multicentre randomized phase 2 DIRECT trial	Nature Communications	June 2020	902	46
47	Effect of three decades of screening mammography on breast cancer incidence	New England Journal of Medicine	November 2012	902	830
48	Effect of a scalp cooling device on alopecia in women undergoing chemotherapy for breast cancer	JAMA	February 2017	884	97
49	Use of molecular tools to identify patients with indolent breast cancers with ultralow risk over 2 decades	JAMA Oncology	November 2017	882	39
50	Breast-cancer risk in families with mutations in PALB2	New England Journal of Medicine	August 2014	879	494

The highest and lowest AASs in the top 50 article list were 3,619 and 879, respectively, while the median AAS was 1,063. The highest numbers of articles were published in 2017 (n = 9) and 2020 (n = 9). The top 50 articles were published in 28 different journals; eight articles were published in the New England Journal of Medicine, followed by the Journal of the American Medical Association (JAMA) with four articles (Table 3). All of the articles were published in journals in the highest impact factor (IF) quartile.

**Table 3:** Journals with top 50 articles ranked according to the Altmetric Attention Score. * Impact Factor, 2020 Journal Impact Factor, Journal Citation Reports, Clarivate, 2022. ** 2022 Scimago Journal and Country Rank. JAMA: Journal of the American Medical Association; JNCI: Journal of the National Cancer Institute; N/A not available

Rank	Journal name	Number of articles	Impact factor*	Impact factor ranking**	H-index**
1	New England Journal of Medicine	8	91.2	Q1	1,030
2	JAMA	4	56.2	Q1	680
3	JAMA Oncology	3	31.7	Q1	99
3	Nature	3	49.9	Q1	1,226
3	Nature Genetics	3	38.3	Q1	573
6	British Medical Journal	2	39.8	Q1	429
6	Cancer	2	6.8	Q1	304
6	International Journal of Cancer	2	7.3	Q1	234
6	JAMA Internal Medicine	2	21.8	Q1	342
6	Lancet Oncology	2	41.3	Q1	324
6	The Lancet	2	79.3	Q1	762
7	Annals of Internal Medicine	1	25.3	Q1	390
7	Breast Cancer Research	1	6.4	Q1	149
7	CA: A Cancer Journal for Clinicians	1	508.7	Q1	168
7	Cancer Cell	1	31.7	Q1	335
7	Carcinogenesis	1	4.9	Q1	204
7	Current Developments in Nutrition	1	N/A	Q1	14
7	Food & Chemical Toxicology	1	6	Q1	172
7	Health Affairs	1	6.3	Q1	178
7	International Journal of Epidemiology	1	7.1	Q1	208
7	JAMA Network Open	1	8.4	Q1	39
7	JNCI	1	13.5	Q1	356
7	Journal of Clinical Oncology	1	44.5	Q1	548
7	Molecular Cancer Therapeutics	1	6.2	Q1	173
7	Nature Communications	1	14.9	Q1	365
7	NPJ Precision Oncology	1	8.2	N/A	N/A
7	PLoS One	1	3.2	Q1	332
7	Preventive Medicine	1	4	Q1	169

The top-ranked article (#1 in Table 2) reported a deep learning model (artificial intelligence) for identifying breast cancer in screening mammograms. In that study, the authors presented an artificial intelligence system that outperforms radiologists on a clinically relevant breast cancer identification task. Classification according to subject categories is shown in Table 4. The most frequent top three subjects in the top 50 articles were “treatment and management” (n = 12), “risk factors for breast cancer” (n = 11), and “breast cancer screening” (n = 11). The most frequently mentioned subject in the treatment and management category (#2, #9, #22, and #49) was the role of multi-gene tests in identifying recurrence and adjuvant treatment in operated patients with early-stage breast cancer. There were two publications (#4 and #17) in the treatment and management category mentioning the role of tumor-infiltrating lymphocytes in the treatment of breast cancer. One of these articles (#4) was a case report which is the only case report included in the top 50 list. It is worth noting that half of the top 10 articles were about treatment and management. There were 11 articles concerning the risk factors of breast cancer, while the leading risk factors drawing interest were hair care product use (#3 and #30), menopausal hormone therapy (#12 and #27), and specific dairy foods (#37 and #42). In the top 50 list, 11 articles were noted regarding mammography which has been in use for many years for breast cancer screening. Mammography screening effectiveness (#7, #34, and #47) and impact on mortality (#15, #29, and #39) were investigated in more than half of the articles regarding screening. Only one article in the top 50 list was about coronavirus disease 2019 (COVID-19), and the subject in this article (#41) was the impact of COVID-19 on screening test volume. In April 2020, a 96% decrease was reported in breast cancer screening tests. The other subject categories included in the top 50 list were the pathogenesis of breast cancer (n = 7), breast cancer prevention (n = 3), quality of life (n = 2), diagnosis (n = 2), psycho-oncology (n = 1), and breast cancer statistics (n = 1) (Table 4).

**Table 4:** Numbers of articles with top 50 Altmetric Attention Scores according to subject categories.

Subject category		Number of articles
Treatment and management		12
	Breast cancer multigene testing for a recurrence score	4
	Tumor-infiltrating lymphocytes (TILs)	2
	Dietary supplement	1
	Investigational new drug study (BOS172722)	1
	Intraoperative radiation therapy (IORT) for early breast cancer	1
	Atezolizumab in advanced triple-negative breast cancer	1
	Early mortality after systemic anticancer treatment	1
	Fasting mimicking diet and neoadjuvant chemotherapy toxicity	1
Risk factors for breast cancer		11
	Hair care product use	2
	Menopausal hormone therapy	2
	Specific dairy foods	2
	Modifiable and nonmodifiable risk factors	2
	Hormonal contraception	1
	Mistimed eating patterns	1
	Mutations in PALB2	1
Breast cancer screening		11
	Mammography screening effectiveness	3
	Impact on mortality	3
	Evaluation of an artificial intelligence	1
	Guideline	1
	Cost-effectiveness analysis	1
	Evaluation of tomosynthesis	1
	COVID-19 impact on screening test volume	1
The pathogenesis of breast cancer		7
Breast cancer prevention		3
	Mediterranean diet	1
	Sustained weight loss	1
	Dietary isoflavone	1
Quality of life		2
	Prevention of chemotherapy-induced alopecia (scalp cooling)	2
Diagnosis		2
	Pigeons as trainable observers	1
	Assessment of machine learning	1
Psycho-oncology		1
	Postdiagnosis social networks	1
Breast cancer statistics		1

The designs and evidence levels of the articles in the top 50 list are shown in Table 5. As shown in the table, there were 43 articles in the clinical research category, four in the experimental animal studies category, and three in the tumor cell culture studies category. In the top 50 list, the number of articles with a level of evidence of 1, 2, 3, and 4 was 12, 17, 17, and 4, respectively. The top five articles with the highest number of citations are ranked at #28, #40, #35, #22, and #47 in Table 2. With 1,562 citations, the most highly cited publication (#28) was an article titled “Atezolizumab and Nab-Paclitaxel in Advanced Triple-Negative Breast Cancer” published in the New England Journal of Medicine in 2018.

**Table 5:** Study design and levels of evidence by SIGN of the top 50 articles. SIGN: Scottish Intercollegiate Guidelines Network

Study type and subtype		Level of evidence	Number of articles
Clinical research			
	Meta-analysis	1	1
	Randomized controlled trial	1	11
	Prospective cohort study	2	11
	Observational descriptive study	3	1
	Cross-sectional study	3	5
	Retrospective cohort study	3	7
	Retrospective comparative study	3	2
	Case report	3	1
	Expert opinion (editorial or letter)	4	3
	Expert committee report	4	1
Experimental animal study			
	Prospective comparative study	2	3
	Observational study	3	1
Tumor cell culture study			
	Prospective comparative study	2	3

According to the top 50 list, the correlation between AAS and citation was not significant (r = 0.11, p = 0.244). Additionally, there was no correlation between AAS and the number of years post-publication (r = -0.09, p = 0.506). As expected, there was a significant positive correlation between citation and the number of years post-publication (r = 0.558, p < 0.001). According to the stratification of the top 50 list articles based on the level of evidence, the median AAS was 1,112.5 (IQR = 929.5-1,444) for level 1, 1,052 (IQR = 929.5-1,456) for level 2, 1,012 (IQR = 932-1,475) for level 3, and 1,167 (IQR = 921.5-1,334.5) for level 4, without any significant difference between the groups (p = 0.994).

## Discussion

Cancer is a major disease burden worldwide. Breast cancer is the most common cancer diagnosed among US women (excluding skin cancers) and is the second leading cause of cancer death among women after lung cancer [8]. Breast cancer mortality has declined significantly in recent years. This is in line with increased early detection rates as a result of the implementation of national population-based mammography screening programs and more effective adjuvant therapy [9]. This study revealed the aspects regarding which the academic world and society interacted more frequently on social platforms about breast cancer while uncovering the trending publications. To our knowledge, this study is the first to assess the online attention that articles published in the field of breast cancer received.

The journals that published articles receiving the most attention on social media were the New England Journal of Medicine (n = 8) and JAMA (n = 4). These two journals are not specifically oncology journals. Table 3 shows that only 34% (n = 17) of the top 50 articles were published in cancer-specific journals. Moreover, 70% (n = 35) of the top 50 articles were published in journals with an IF of >10. It can be said that the articles receiving the most attention in society regarding breast cancer were published in high IF general medicine journals instead of oncology-specific journals. The activity level of the journals on social media may have had an impact in addition to the research topic that the academic publications handle. Future studies can investigate this relevance. A study conducted in 2019 by Kim et al. evaluated the top 50 articles with the highest AAS in the field of stroke [10]. According to the results of this study, several high IF journals such as the New England Journal of Medicine, JAMA, and The Lancet are included in the top 50 stroke list. On the other hand, those journals did not receive the highest rankings or included the largest numbers of published articles. If an academic journal prioritizes social media management more, it can have a higher AAS. Investigating this subject in the future with altmetric studies can provide significant contributions to the literature. It should be kept in mind that traditional metrics and the AAS measure different perspectives.

Table 4 demonstrates that many different aspects of breast cancer have been discussed in the top 50 list. A review of this table reveals that articles on diet and cosmetics in breast cancer come into prominence. While the association between diet and the effectiveness and toxicity of chemotherapy was investigated in two articles [11,12], diet and the risk of breast cancer or the prevention of breast cancer were investigated in eight articles. Two articles discussed the association between hair care product use and the risk of breast cancer [13,14]. Moreover, two articles evaluated the efficiency of the use of the scalp cooling device in chemotherapy for breast cancer in reducing alopecia [15,16]. As a result, the association between breast cancer and diet and cosmetics attracts public attention on social media. The top 50 list did not include any articles regarding breast cancer surgery which is the primary treatment for early-stage breast cancer and has been improving with more effective techniques over the years [17,18]. Furthermore, there were no scientific articles in the top 50 list involving major treatment agents such as CDK4/6 inhibitors or HER-2 blockage (trastuzumab, pertuzumab), which are among the revolutionary treatments that significantly prolonged survival in metastatic breast cancer [19,20]. This suggests that there are some perspective differences between academic papers and general social media. Clinicians are interested in various topics such as the pathogenesis of the disease, treatment guidelines, new diagnostic tools, surgery techniques, and new drugs that members of the general public are not interested in.

The data in Table 5 provide notable information about the level of evidence of articles on breast cancer that attracted more attention on social media. In the top 50 list, the number of articles with the lowest level of evidence (SIGN Level 4) was only four. Articles with levels of evidence of 1 and 2 which were analytical trials constituted 58% of the entire group. Although the level of evidence is a significant parameter for scientists, society may not have sufficient knowledge about the level of evidence or design of scientific publications. This may cause the information with a low level of evidence to become popular and get disseminated on social media. A classification of the articles in the top 50 list according to the four levels of evidence did not produce any statistically significant difference between the groups in terms of median AASs. Additionally, no significant correlation was noted between the numbers of citations and AASs. Similar to our study, a study conducted by Celik et al. involving a correlation analysis of citations and AAS for the top 50 articles on “cancer” did not demonstrate any correlation [21]. Therefore, it can be concluded that the top 50 list includes higher number of articles on breast cancer with higher levels of evidence. The lack of any correlation between the number of citations and AAS once again emphasizes the fact that altmetric analysis assesses different aspects of articles compared to traditional citation analysis and that the interest in scientific studies may differ among the academy and the social media.

The limitation of this study is that the AAS is a relatively new tool. When assessing study results, the absence of AAS in studies published prior to 2011 should be taken into account. The study’s inclusion of 50 articles is another limitation. Instead of 50 articles, choosing 100 or 200 would have enhanced the study’s power.

## Conclusions

One of the novel measures of citations in social media is AAS analysis. This study provides useful information about the impact of the top 50 breast cancer articles in both academia and social media. The AAS seems to be a more reliable assessment of public perception of breast cancer. Finally, we propose that combining the AAS and traditional metrics may provide a more detailed description of scientific research output.
